# Clinical success of guided tissue regeneration for treating vertical bone and furcation defects in dogs

**DOI:** 10.3389/fvets.2023.1247347

**Published:** 2023-08-30

**Authors:** Bonnie L. Lee, Jason Soukup, Aaron Rendahl, Stephanie Goldschmidt

**Affiliations:** ^1^Department of Veterinary Clinical Sciences, College of Veterinary Medicine, Dentistry and Oral Surgery Service, University of Minnesota, St. Paul, MN, United States; ^2^Department of Surgical Sciences, School of Veterinary Medicine, Dentistry and Oral Surgery Service, University of Wisconsin, Madison, WI, United States; ^3^College of Veterinary Medicine, Statistics and Informatics Service, University of Minnesota, St. Paul, MN, United States; ^4^Department of Surgery and Radiologic Sciences, Veterinary Medical Teaching Hospital, Dentistry and Oral Surgery Department, University of California, Davis, Davis, CA, United States

**Keywords:** guided tissue regeneration, GTR, periodontal disease, infrabony defect, vertical bone loss, furcation, bone graft, barrier membrane

## Abstract

This study evaluated the clinical success rate of guided tissue regeneration (GTR) for treating advanced periodontal disease in a large canine cohort. A total of 112 GTR procedures performed from 2003–2021 were retrospectively evaluated, including pre- and post-treatment (3–12 months) periodontal probing depths of 104 treated teeth, dental radiographs of 73 treated teeth, and both diagnostic modalities in 64 treated teeth. Probing depth, radiographically apparent bone height, bone graft material, barrier membrane material, and tooth extraction adjacent to the GTR site were investigated as factors affecting success. Vertical bone defects were evaluated separately from furcation defects. GTR was clinically successful, defined as objective improvement in probing depth, objective decrease in radiographic vertical bone defect, and subjective radiographic gain in bone height in 90.3% of vertical bone defects. Success was significantly associated with the magnitude of initial probing depth and the type of barrier membrane used. GTR was clinically successful, defined as objective improvement in furcation probing and subjective radiographic improvement of the bone in the furcation in 22.2% of furcation defects. When F3 lesions were excluded, GTR was successful in 64.3% of furcation defects. GTR is an appropriate treatment to maintain teeth in the oral cavity of dogs with proper client counseling and patient selection, but it is most likely to be successful in vertical defects.

## Introduction

Periodontal disease is highly prevalent within the canine population, affecting up to 85% of dogs by 3 years of age (1). Teeth affected by moderate to severe periodontitis have historically been treated with exodontia (2). However, despite the presumption that edentulous dogs can maintain a good quality of life, maintaining teeth, particularly the strategic canine and carnassial teeth, is advantageous (3). This allows functional use of the teeth for prehension and mastication as well as the natural cleaning action of occlusal pairs (4). The patient also avoids the potential complications of exodontia, including, but not limited to, soft tissue trauma, nerve damage, root tip fracture with displacement, and excess bone removal with possible secondary bone fracture (2).

Advanced periodontal treatments have been translated from human dentistry to provide alternative treatment options for exodontia in canine patients. Advanced periodontal treatment options include open root planing (RP/O), RP/O with local delivery of antimicrobial agents or implant placement, and guided tissue regeneration (GTR). GTR may be indicated in cases of moderate to severe periodontitis, especially when there is an infrabony defect. GTR involves RP/O (debridement of the root surface exposed by a mucogingival flap), followed by barrier membrane placement prior to mucogingival flap closure to cover the defect and prevent downward migration of gingival epithelial and connective tissues along the cemental wall (5, 6). Prior to barrier membrane placement, a bone graft can be placed within the defect to maintain space for clot stabilization (5, 6). When utilized appropriately, GTR ideally facilitates periodontal regeneration or otherwise periodontal repair, thereby maintaining the affected tooth in a functional and comfortable state within the oral cavity.

Advanced periodontal therapies, including GTR, are the standard of care for humans rather than exodontia. However, the success of GTR in canine patients is unknown, limiting our ability to routinely incorporate this into veterinary clinical practice. Case reports and small case series have documented clinical success in all instances as a proof-of-concept, but none provide robust data due to small case numbers (Supplementary Table 1) (7–12). This study aimed to establish the clinical success of GTR in a large canine cohort and identify factors associated with clinical success.

## Materials and methods

Dogs treated with GTR from 2003–2021 were identified by searching the electronic medical records (EMR) from two academic institutions. The EMR at the University of Minnesota Veterinary Medical Center was queried using codes for the following products: “Periomix,” “Consil,” “Ossiflex,” and “Doxirobe,” as these are products currently and historically used for GTR at this institution. Keyword search for “guided tissue regeneration” or other terms was unavailable due to limitations of the EMR software used at this institution. The EMR at the University of Wisconsin Veterinary Medical Center was queried using the following keywords: “guided tissue regeneration” and “GTR.” All resultant records (paper and electronic) were reviewed manually to ensure that patients met the inclusion criterion.

The inclusion criterion was GTR performed with at least one anesthetized follow-up examination within 3–12 months of the procedure. Follow-up examination had to include either periodontal probing or dental radiographs. Not having both diagnostic modalities at the follow-up was not an exclusion criterion. However, data that had both diagnostic modalities were prioritized and presented as the primary evaluator of clinical success. Teeth treated with GTR should always be evaluated with both pre- and post-treatment periodontal probing and dental radiographs.

Clinical patient data acquired included age, breed, gender, weight, treated tooth, specific treatment location on the tooth, bone graft material, barrier membrane material, presence of dentigerous cyst within the GTR site, and if teeth adjacent to the GTR site were extracted. Specific tooth treatment locations were recorded as follows: mesial, mesiobuccal, buccal, mesiopalatal, palatal, distal, furcation, or no remarks. Bone graft materials were categorized as autografts, allografts, or alloplasts (Table 1). Barrier membrane material was also recorded.

**Table 1:** Bone graft materials used in GTR treatment in this study.

**Bone graft**	**Definition**	**Properties**	**Products**
Autograft	Bone collected from the same patient in which it was used	Osteogenic	Osseous coagulum
		Osteoinductive	
		Osteoconductive	
Allograft	Demineralized bone matrix with cancellous bone chips from a different individual than in which it was used	Osteoinductive	Periomix (Veterinary Transplant Services Inc., Kent, WA, USA) Fortigen-P (Veterinary Transplant Services Inc., Kent, WA, USA)
		Osteoconductive	
Alloplast	Not made of bone	Osteoconductive	Consil (Nutramax Laboratories Veterinary Sciences Inc., Lancaster, SC, USA) Synergy (Veterinary Transplant Services Inc., Kent, WA, USA)

Data were collected from the pre- and post-treatment dental charts and dental radiographs. Radiographs were evaluated in an open-source platform for biological image analysis (13). Radiographs were evaluated separately by a resident in dentistry and oral surgery (BLL) and two board-certified dentists (SG, JS). Data from vertical bone defects and furcation defects treated with GTR were collated and analyzed separately.

### Vertical bone defects

**Figure 1:**
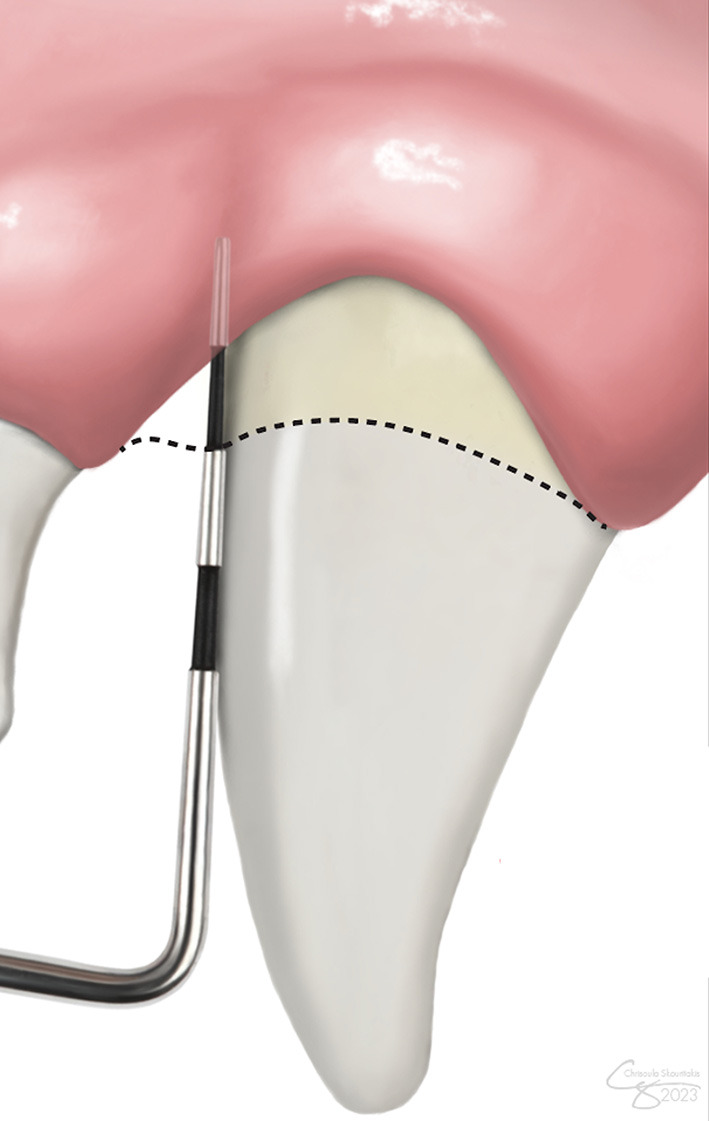
Clinical attachment level (CAL) measurement. CAL accounts for both gingival recession and periodontal probing depth. The level of the cementoenamel junction is demarcated by the dashed line.

**Figure 2:**
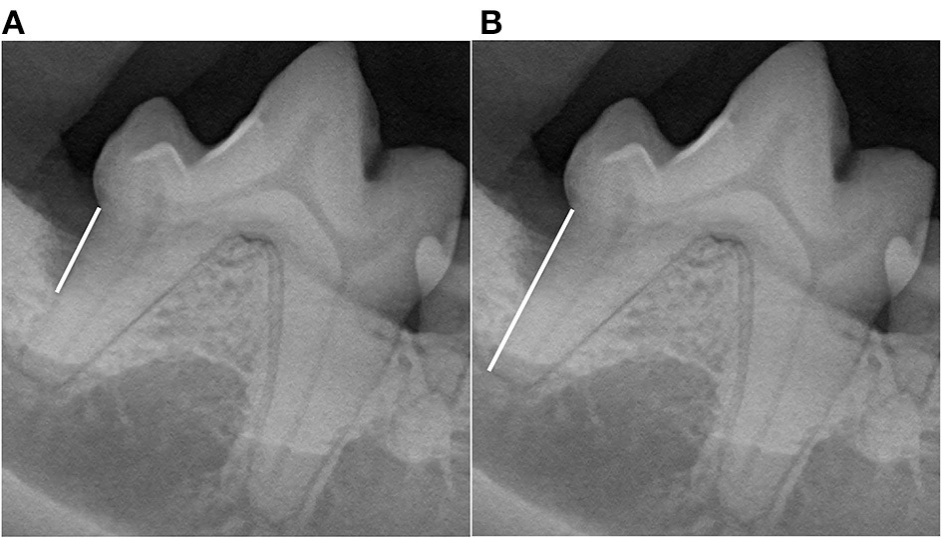
Vertical bone defect ratio (VBDR). Measurements were performed using the line tool to draw lines (white lines) to measure both **(A)** root length, defined as the distance from the cementoenamel junction to the apex, and **(B)** the vertical bone pocket depth, defined as the distance from the cementoenamel junction to the most apical aspect of bone loss at the GTR site. The bone pocket depth ratio was generated as the ratio of the vertical bone pocket depth to the root length.

Pre-treatment data collected from the dental chart included clinical attachment level (CAL) in millimeters (mm), defined as periodontal probing depth plus gingival recession, at the GTR site (Figure 1). The ratio of the vertical bone defect:total root length (vertical bone defect ratio-VBDR) was calculated to allow comparison with the post-treatment radiograph and negate the effects of angulation on defect appearance. Measurements were performed using the line tool in an open-source platform for biological image analysis (13). Lines were drawn to measure both the vertical bone pocket depth, defined as the distance from the cementoenamel junction to the most apical aspect of bone loss, and the total root length, defined as the distance from the cementoenamel junction to the apex (Figure 2). All measurements were performed by a resident in dentistry and oral surgery (BLL).

Post-treatment data were collected as CAL and VBDR from the dental charts and dental radiographs, respectively. The success of the procedure was evaluated subjectively and objectively. Five categories of success were evaluated: objective attachment gain, objective bone improvement, objective success, subjective success, and combined success, as defined below.

Objective attachment gain was calculated by comparing the CAL on the pre- and post-treatment dental charts. Of note, if there were no remarks regarding probing depth or gingival recession on an anesthetized follow-up examination, it was assumed to be a normal gingival sulcus with a maximal depth of 3 mm. Success was defined as an attachment gain of ≥1 mm.

Objective bone improvement was calculated by comparing the VBDR between pre- and post-treatment radiographs. VBDR was categorized as improved, static, or worse. We allowed for a 10% variation for this categorization. Therefore “improved” was defined as a post-treatment VBDR <90% of pre-treatment VBDR; “worse” was defined as a post-treatment VBDR >110% of the pre-treatment VBDR; and “static” was defined as a post-treatment VBDR that fulfilled neither of the above parameters by being within ±10% of the pre-treatment VBDR. This 10% variation was allotted to account for human measurement error.

Objective success was defined as improvement in both CAL and VDBR. It was reported if both improved, both failed, or if there was no consensus when one improved while the other failed.

**Figure 3:**
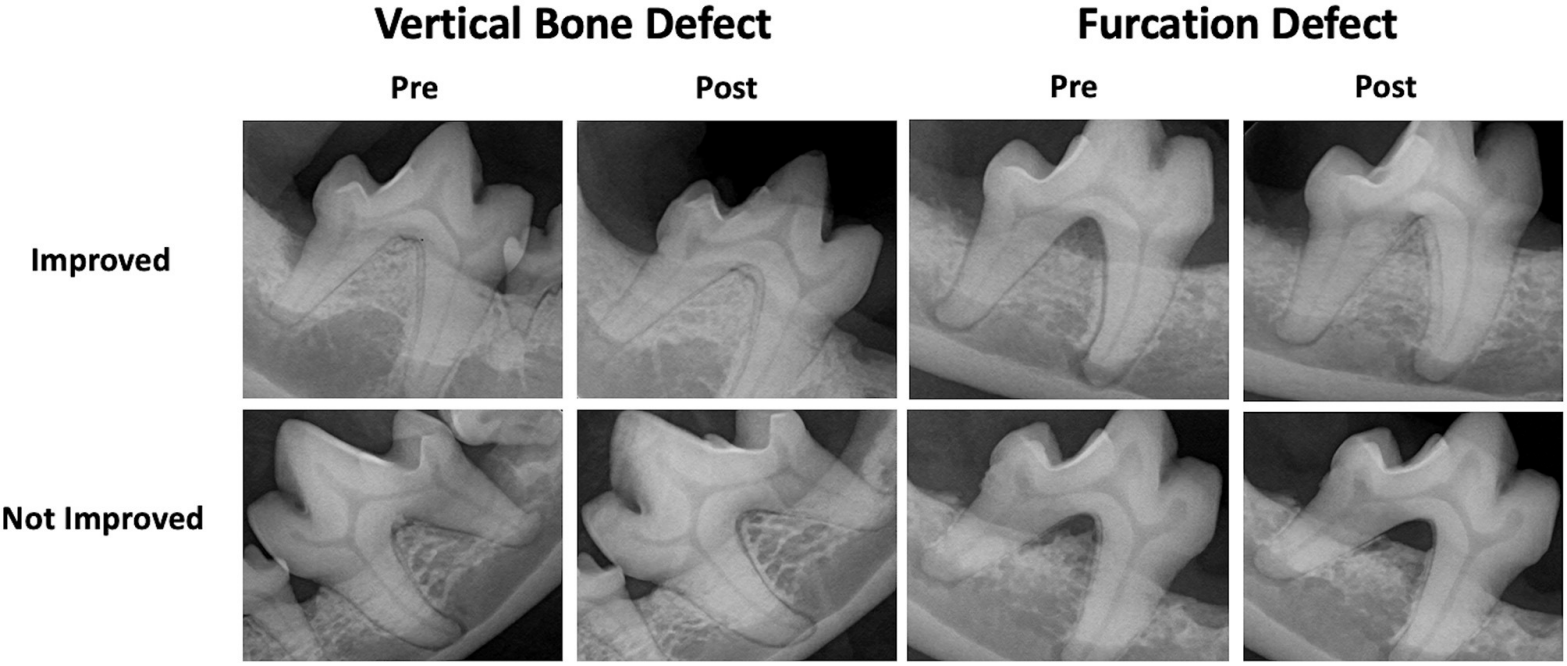
Subjective GTR success. Examples of pre- and post-treatment radiographs of vertical bone and furcation defects treated with GTR resulting in subjective improvement and failure.

Subjective success was determined by comparing pre- and post-treatment radiographs. Images were reviewed by a resident in dentistry and oral surgery (BLL) and two board-certified dentists (SG, JS). Each reviewer categorized the GTR as clinically improved or failed (stagnant or worse) (Figure 3). For analysis, the majority (2/3 reviewers) was followed.

Combined success was defined as both subjective and objective success. It was reported if they both improved, both failed, or no consensus when one improved while the other failed.

### Furcation defects

Pre-treatment data collected from the dental chart included the furcation involvement (F1–F3) at the GTR site, defined as per the American Veterinary Dental College (14). Three parameters of success were evaluated from the post-treatment dental charts and radiographs: objective success, subjective success, and combined success, as defined below.

Objective success was determined by comparing the stage of furcation involvement on the pre- and post-treatment dental charts at the GTR site. Furcation involvement that improved to a normal physiologic furcation (normal or no remarks on the chart) was considered resolved, while furcation involvement that improved by at least one stage was considered improved. Success was defined as either resolved or improved furcation involvement. Furcation involvement that remained the same was considered static, except in the case of F3, where it was marked as not improved. Failure was defined as static or not improved. F3 is the final stage of furcation involvement, and thus we were unable to accurately assess any possible worsening for cases of F3.

Subjective success was determined by comparing pre- and post-treatment radiographs. Each reviewer categorized the GTR as clinically improved or failed (stagnant or worse) (Figure 3). For analysis, the majority (2/3 reviewers) was followed.

Combined success was defined as both objective and subjective success. It was reported if they both improved, both failed, or no consensus when one improved while the other failed.

### Statistical analysis

Categorical risk factors and outcomes were reported in counts and percentages, and the association was assessed using Fisher's exact test. Categorical risk factors and numerical outcomes (or the reverse) were reported in the median, quantiles, and max/min, and the association was assessed using the Kruskal-Wallis test. For numerical risk factors and outcomes, Spearman's correlation was reported, and it was also used to test for association. For all tests of association, observations with missing data for those variables were removed, and levels of categorical factors with three or fewer observations were not included. When several risk factors were significant, the association was explored further using multiple linear regression. Models with all terms were first fit and compared with a more parsimonious model using only significant terms. The preferred model was then used to estimate average effects due to each term and predicted values and prediction intervals for individuals with selected characteristics.

For both types of defects, risk factors of interest were institution, bone graft material, barrier membrane material, if there was a dentigerous cyst present within the GTR site, and if teeth adjacent to the GTR site were extracted. For vertical defects, pre-treatment CAL and pre-treatment VBDR were also of interest, and for furcation defects, pre-treatment furcation involvement was also of interest.

## Results

A query of the EMR revealed that 140 patients had GTR performed from 2003–2021. However, 62.3% (33/53) of patients from the University of Minnesota and 54.0% (47/87) from the University of Wisconsin were lost to follow-up. Combined, 57.1% (80/140) of patients that had GTR performed did not return for evaluation within the 12 months following treatment.

A total of 112 GTR procedures performed in 54 patients were included in the analysis of vertical bone defect and furcation defect cases collectively. Of these, 92.8% (104/112) had dental charts available for evaluation, 65.2% (73/112) had diagnostic dental radiographs available for evaluation, and 57.1% (64/112) had dental charts and diagnostic dental radiographs available for evaluation.

The patient population was 50% (27/54) castrated males, 5.6% (3/54) intact males, and 44.4% (24/54) spayed females. The mean (range) age was 8 (0.5−13.75) years. Small breeds were more common, and the mean (range) weight was 11.2 (2.2–42.3) kg. The most common breeds included Terriers at 20.4% (11/54), Dachshunds at 11.1% (6/54), Spaniels at 7.4% (4/54), toy/miniature Poodles at 7.4% (4/54), and Chihuahua/mix at 7.4% (4/54). Other breeds included Shih Tzu/mix (3/54), Retriever/mix (3/54), Shepherd (3/54), miniature Schnauzer (2/54), Bichon Frise/mix (2/54), Hound (2/54), Pug (2/54), Vizsla (1/54), Portuguese water dog (1/54), Maltese (1/54), Papillon (1/54), American Eskimo dog (1/54), Shiba Inu (1/54), Border Collie (1/54), and Boxer (1/54).

**Figure 4:**
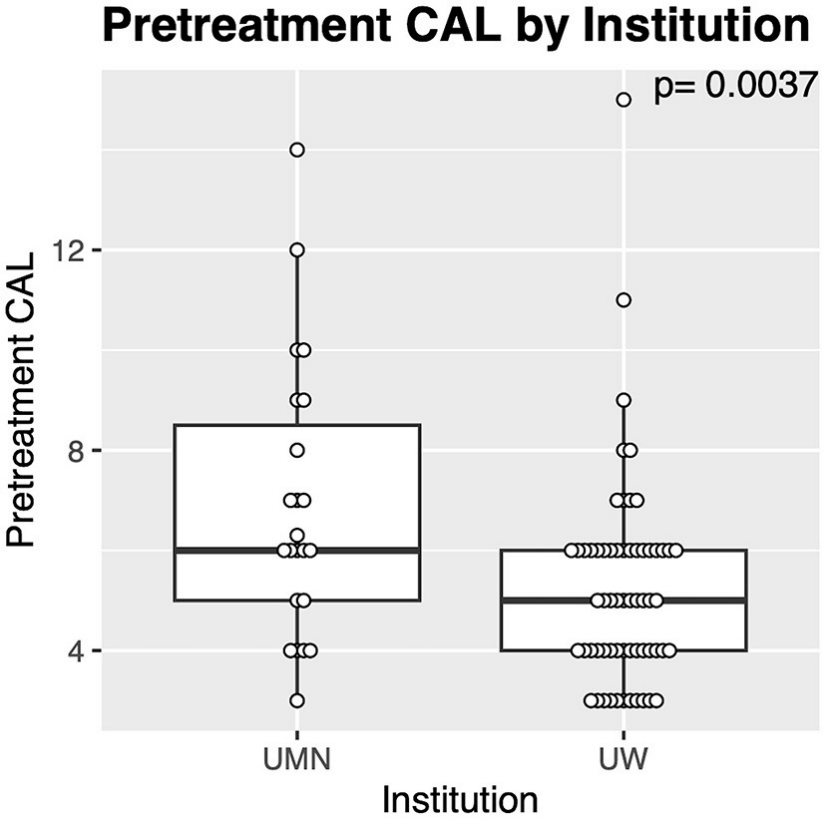
Differences in teeth treated with GTR between institutions. The cases from the University of Minnesota (UMN) had significantly greater pre-treatment CAL compared with those from the University of Wisconsin (UW).

There was no significant difference between the patient population or teeth treated between the two institutions. However, pre-treatment CAL was significantly (*p* = 0.0037) greater for vertical bone defects at the University of Minnesota Veterinary Medical Center (Figure 4). Allografts were also significantly (*p* = 0.014) more common at the University of Minnesota Veterinary Medical Center 95.7% (22/23) compared to the University of Wisconsin Veterinary Medical Center 65.6% (40/61). The same barrier membranes were used at both institutions. When analyzed at the tooth level, there were significantly (*p* = 0.0003) more furcation defects from males at the University of Minnesota Veterinary Medical Center (100%, 20/20), than at the University of Wisconsin Veterinary Medical Center (28.6%, 2/7).

### Vertical bone defects (*n =* 85)

**Figure 5:**
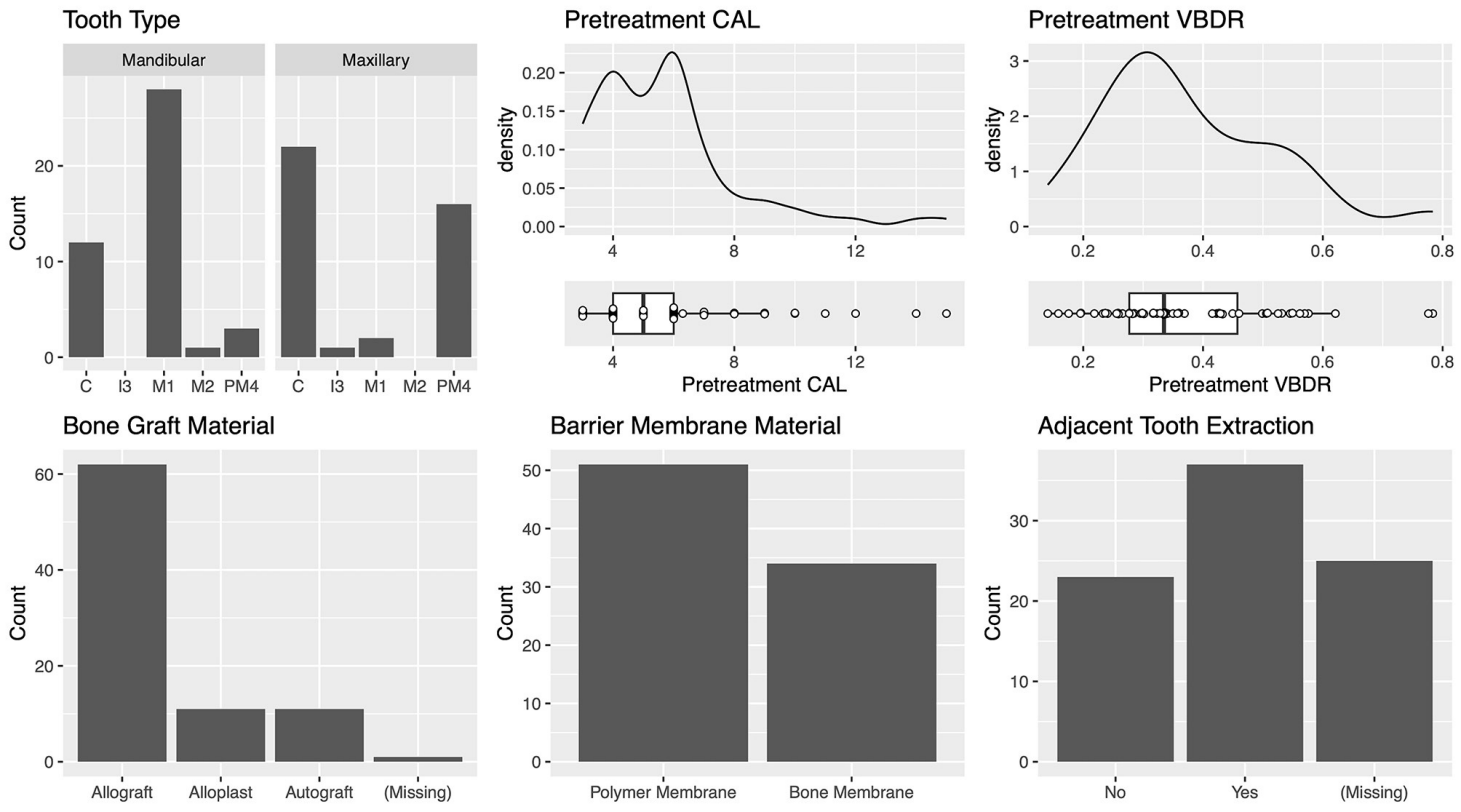
Characteristics of the teeth with vertical bone defects treated with GTR.

The most commonly treated teeth were the mandibular first molar, maxillary canine, and maxillary fourth premolar (Figure 5). For the teeth most commonly treated, the vertical bone defect locations were described in most cases. The defect locations for the mandibular first molar were mesial 42.1% (8/19) and distal 57.9% (11/19). The defect locations for the maxillary canine were palatal 41.2% (7/17), mesiopalatal 29.4% (5/17), and mesial 29.4% (5/17). The defect locations for the maxillary fourth premolar were distal 27.3% (3/11), mesial 36.3% (4/11), mesiobuccal 9.1% (1/11), mesiopalatal 18.2% (2/11), and palatal 9.1% (1/11). The defect locations for the mandibular canine were distal 25% (2/8), mesial 62.5% (5/8), and mesiopalatal 12.5% (1/8).

The median (range) pre-treatment CAL was 5.0 (3–15) mm, and the median (range) pre-treatment VBDR was 0.33 (0.14–0.78) mm, which is equivalent to approximately one-third of the distance from the cementoenamel junction to the apex, and consistent with periodontal disease stage 3, defined as per the American Veterinary Dental College (Figure 5) (14).

The most commonly used bone graft material was an allograft at 73.8% (62/84) (Figure 5). The two different barrier membranes used were (1) a polymer membrane composed of N-methyl-2-pyrrolidone and poly (DL-lactide) delivery system and 8.5% doxycycline (polymer membrane—Doxirobe Gel, Zoetis, Parsipanny, NJ, USA) in 60% (51/85) of cases; and (2) a canine demineralized freeze-dried cortical bone flexible membrane allograft (bone membrane—Ossiflex Bone Membrane, Veterinary Transplant Services Inc., Kent, WA, USA) in 40% (34/85) of cases. The teeth adjacent to the GTR site were extracted during treatment in 61.7% (37/60) of cases (Figure 5). GTR was performed after enucleation of a dentigerous cyst at the site in 3.3% (2/60) of cases.

### Combined success (*n =* 31)

**Figure 6:**
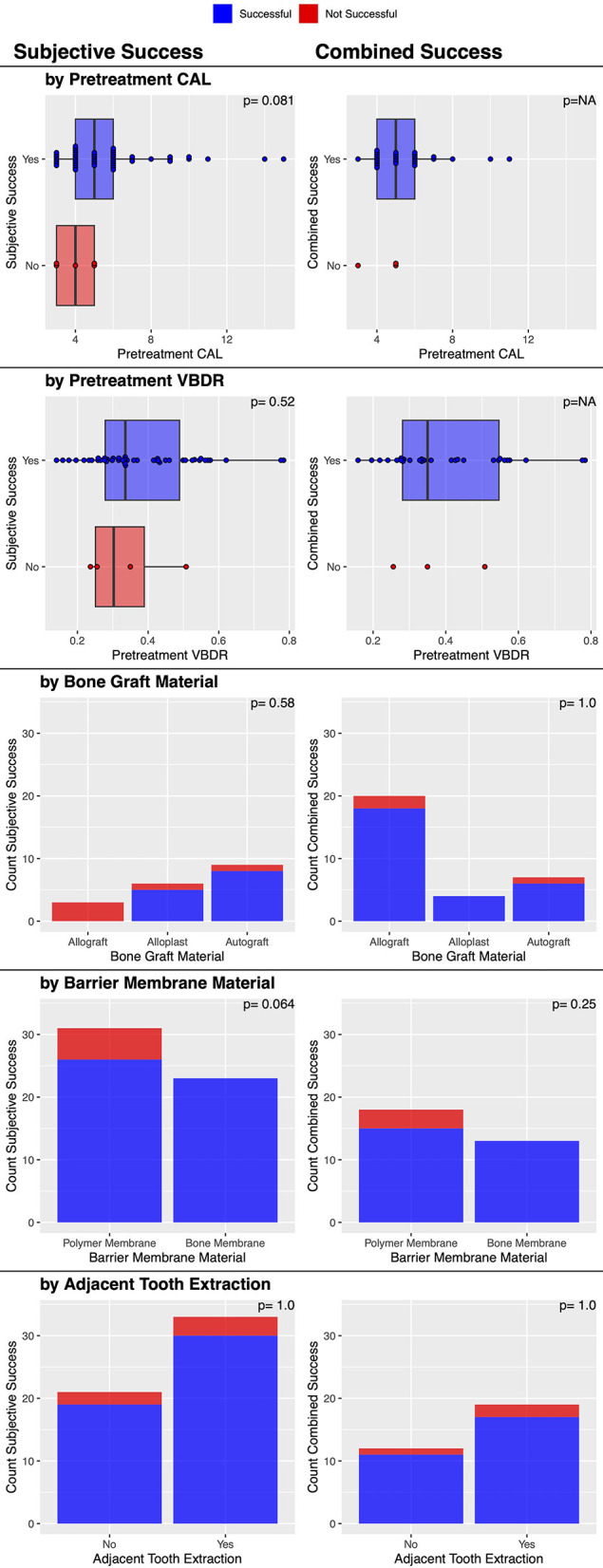
Factors associated with post-treatment subjective and combined clinical success of vertical bone defects treated with GTR.

There was an overall consensus of objective (CAL and VBDR both improved) and subjective improvement in 90.3% (28/31) of cases (Table 2). No factors were significantly associated with this outcome (Figure 6), though the three that failed had used the polymer membrane.

**Table 2:** Definition and success rates of the parameters used to evaluate GTR performed to treat vertical bone defects (*n* = 85).

**Success parameter**	**Evaluation**	**Definition of success**	**Number of cases**	**Percentage success**
1. Objective attachment gain (*n =* 81)	Comparison of pre- and post-treatment CAL	Improved CAL post-treatment	59/81	72.8%
2. Objective bone improvement (*n =* 53)	Comparison of pre- and post-treatment VBDR	Improved VBDR post-treatment	46/53	86.8%
3. Objective success (*n =* 35)	Comparison between 1 and 2	Improved CAL and VBDR post-treatment	31/35	88.6%
4. Subjective success (*n =* 54)	Subjective evaluation of pre- and post-treatment radiographs	Clinically improved	49/54	90.7%
5. Combined success (*n =* 31)	Comparison between 3 and 4	Objective and subjective success	28/31	90.3%

### Objective success (*n =* 35)

**Figure 7:**
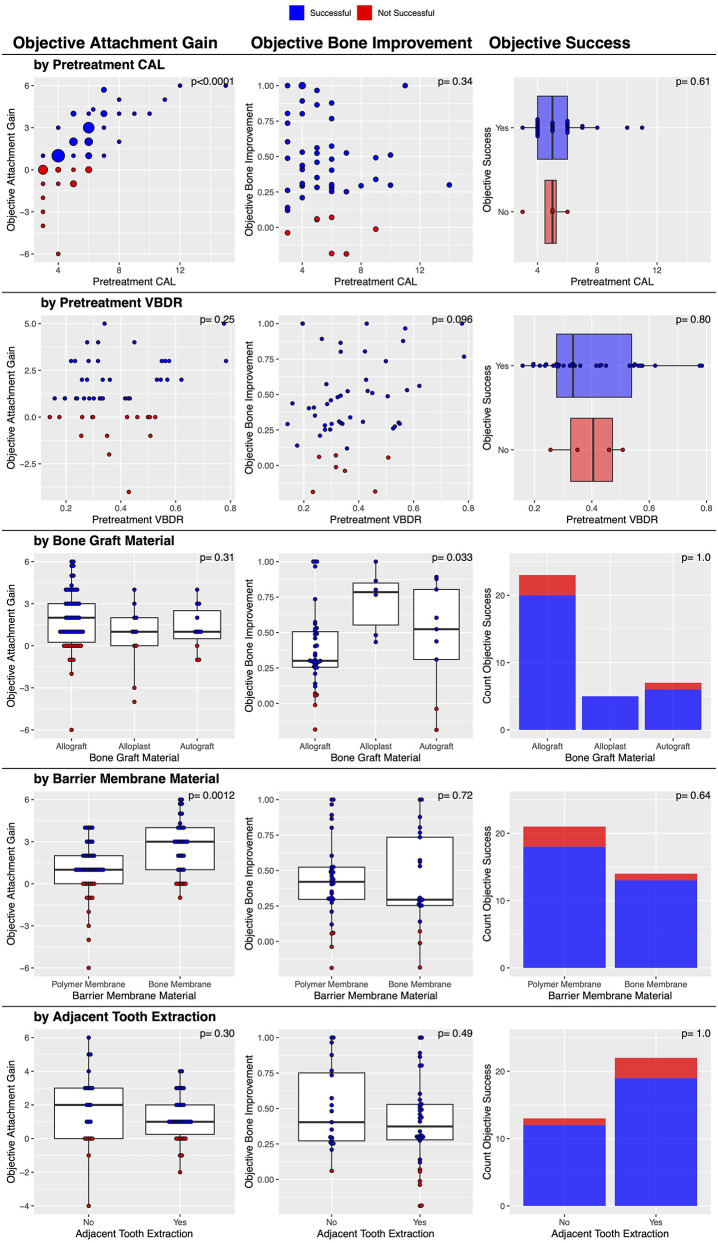
Factors associated with post-treatment objective clinical success of vertical bone defects treated with GTR.

Post-treatment dental charts and radiographs were available in 35 cases. Both diagnostic modalities showed an objective improvement in 88.6% (31/35) of cases (Table 2). No factors were significantly associated with this outcome (Figure 7).

### Objective attachment gain (*n =* 81)

Post-treatment dental charts were available for 81 cases. There was objective attachment gain in 72.8% (59/81) of the vertical bone defects treated with GTR (Table 2). In 11.1% (9/81) of cases, CAL change was zero, and in 16.1% (13/81), CAL worsened. In this study, the median pre-treatment CAL (range) was 5.0 (3–15) mm. The median (range) improvement in CAL was 1.0 (−6–6) mm, suggesting that while there was a median improvement of 1.0 mm, this ranged from worsening by 6 mm to improving by 6 mm.

There was a statistically significant association between the barrier membrane material used and the magnitude of post-treatment attachment gain. The bone membrane resulted in a significantly (*p* = 0.0012) greater attachment gain (a median of 3 mm improvement in CAL) compared to the polymer membrane (a median of 1 mm improvement in CAL). The severity of pre-treatment CAL was also significantly (*p* < 0.0001) associated with the magnitude of post-treatment attachment gain, with a greater initial attachment loss resulting in a significantly greater attachment gain (Figure 7). Finally, the institution was also significantly associated (*p* = 0.0036) with attachment gain; the veterinary center at the University of Minnesota had a median of 3 mm, and the center at the University of Wisconsin had a median of 1 mm. No other factors were significantly associated with this outcome (Figure 7).

The associations with objective attachment gain were explored further using multiple linear regression. First, a model was fit with the institution, bone graft material, barrier membrane material, pre-treatment CAL, pre-treatment VBDR, and extraction of an adjacent tooth. In the model, the relationships with pre-treatment CAL and pre-treatment VBDR were allowed to be non-linear by using a natural spline with two degrees of freedom. In the ANOVA table for this model, only barrier membrane material and pre-treatment CAL were statistically significant, so this parsimonious model was fit and compared to the full model; the full model was not statistically significantly better (*p* = 0.20). Although the institution was significantly associated with the objective attachment gain in the univariate analysis, it was not significant in the multiple linear regression because the institution was also associated with pre-treatment CAL, which better explained the variability in the objective attachment gain.

**Figure 8:**
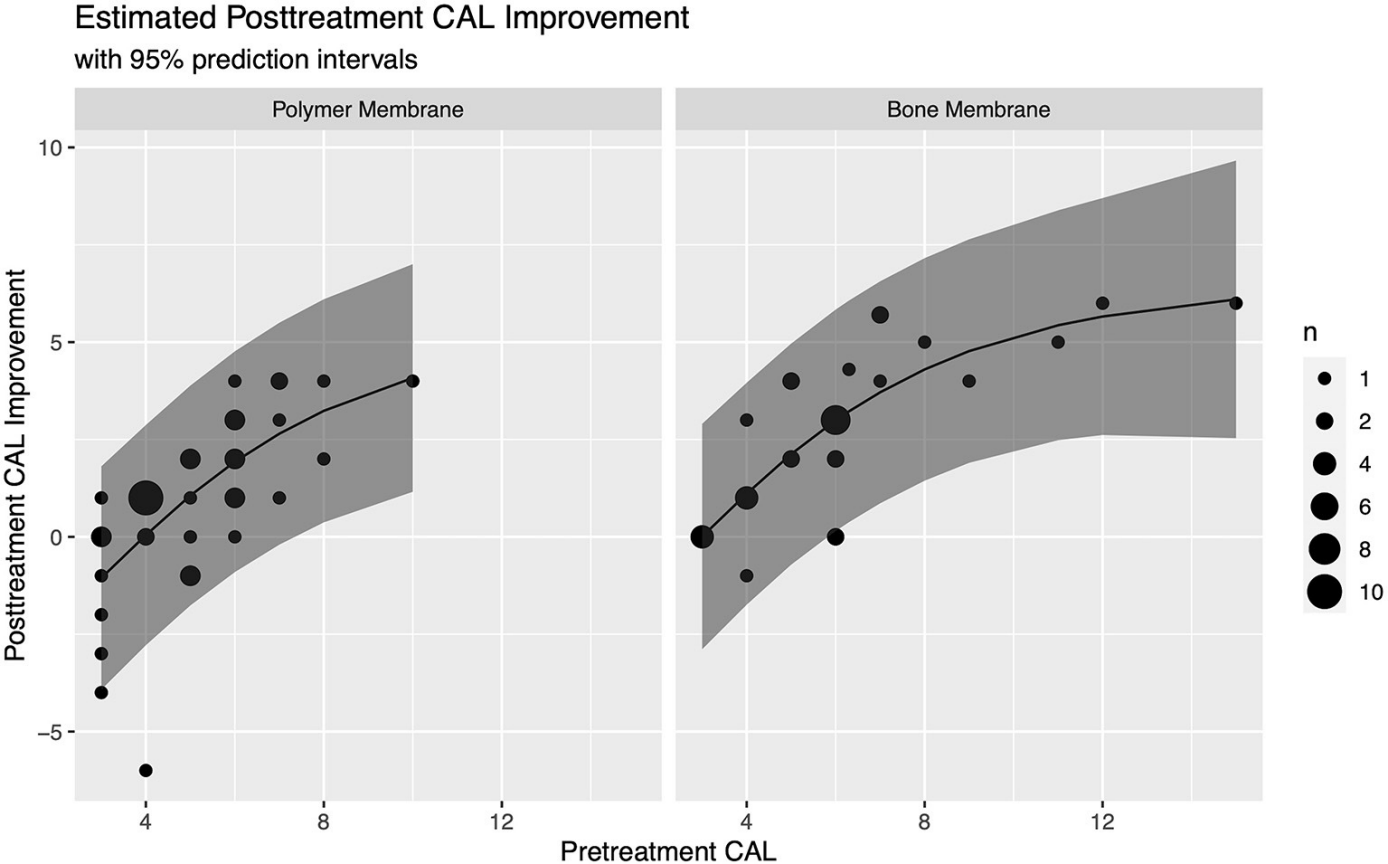
Estimated CAL improvement for vertical bone defects treated with GTR based on barrier membrane material and pre-treatment CAL with 95% prediction intervals.

In this model, the improvement with the bone membrane was found to be an average of 1.06 (95% CI: 0.42–1.71) mm greater than with the polymer membrane, and the improvement for a pre-treatment CAL of 8 mm was found to be an average of 3.2 (95% CI: 2.5–3.9) mm higher than when the pre-treatment CAL was 4 mm. For clinical purposes, not all animals are average, so we reported estimated averages along with the margin of error for a 95% prediction interval for these representative animals to give a sense of the expected variability. For animals with a pre-treatment CAL of 4 mm, we estimated an improvement of 0.04 ± 2.83 mm with a polymer membrane and 1.11 ± 2.85 mm with a bone membrane, while for animals with a pre-treatment CAL of 8 mm, we estimated an improvement of 3.24 ± 2.87 mm with a polymer membrane, and 4.3 ± 2.86 mm with a bone membrane (Figure 8).

### Objective attachment gain (*n* = 53)

Post-treatment dental radiographs were available for 53 cases. There were objective bone improvements in 86.8% (46/53) of the vertical bone defects treated with GTR (Table 2). In 9.4% (5/53) cases, the vertical bone defect remained static, and in 3.8% (2/53), the vertical bone defect worsened. The median change in objective bone improvement was 0.40 mm, which is a 40% decrease in vertical bone defect depth, with a range of −0.19 to 1 mm.

There was a statistically significant association between the type of bone graft material used and the magnitude of post-treatment VBDR improvement. The bone graft material was significantly (*p* = 0.033) associated with post-treatment VBDR improvement, with alloplasts having a median of 0.78, autografts a median of 0.52, and allografts a median of 0.30. No other factors were significantly associated with this outcome (Figure 7). However, when treated as success or/failure, the evidence became weaker (*p* = 0.66) with 100% (6/6) success in alloplasts, 77.8% (7/9) success in autografts, and 86.8% (33/38) with allografts.

### Subjective success (*n =* 54)

There were subjective bone improvements in 90.7% (49/54) of cases (Table 2). No other factors were significantly associated with this success outcome (Figure 6), though it may be of interest that the five that failed used the polymer membrane; 100% (23/23) of cases where the bone membrane was used were successful, while 83.9% (26/31) where the polymer membrane was used were successful (*p* = 0.064).

### Furcation defects (*n =* 27)

**Figure 9:**
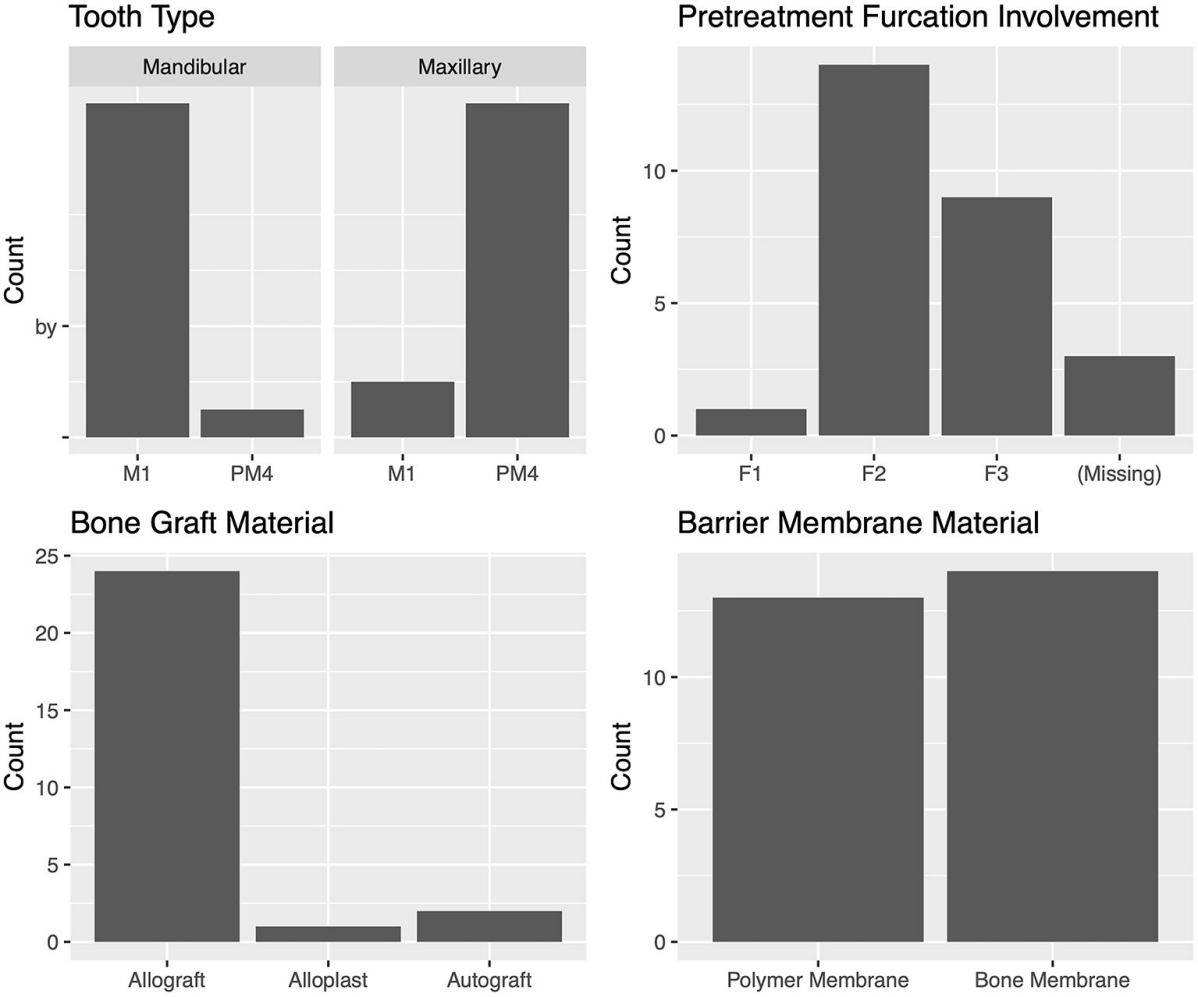
Characteristics of the teeth with furcation bone defects treated with GTR.

The most commonly treated teeth were the carnassial teeth, representing 88.8 % (24/27) of all treated teeth. The distribution of furcation involvement included F1 4.2% (1/24), F2 58.3% (14/24), and F3 37.5% (9/24). The most commonly used bone graft material was an allograft, which was used in 88.9% (24/27) of cases. The polymer membrane was used in 48.1% (13/27) of cases, and the bone membrane in 51.9% (14/27). The teeth adjacent to the tooth treated with GTR were extracted in 30.0% (6/20) of cases (Figure 9). There were no cysts associated with any of the furcation GTR sites.

### Combined success (*n =* 9)

**Figure 10:**
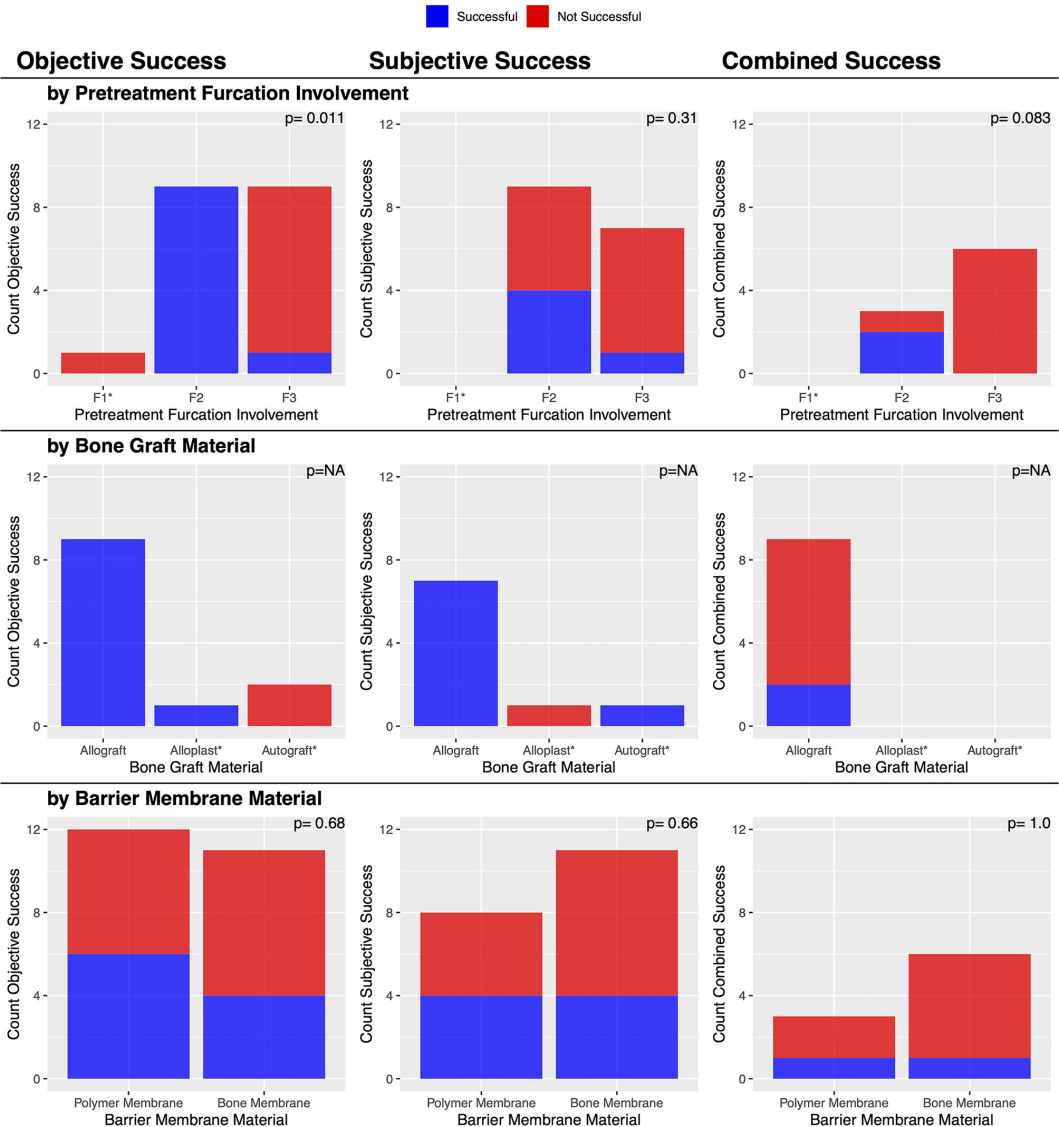
Factors associated with post-treatment clinical success of furcation defects treated with GTR.

There was an overall consensus of both objective (furcation probing) and subjective (radiographic) improvement in 22.2% (2/9) of cases (Table 3). The two successful cases had an initial furcation involvement of F2. There was an overall consensus of failure in 77.8% (7/9) of cases, consisting of 6/6 cases of furcation involvement of F3 and 1/3 cases of furcation involvement of F2 (*p* = 0.083). No other factors were significantly associated with this outcome (Figure 10).

**Table 3:** Definition and success rates of the parameters used to evaluate GTR performed to treat furcation defects (*n* = 27).

**Success parameter**	**Evaluation**	**Definition of success**	**Number of cases**		**Percentage success**	
1. Objective success	Comparison of pre- and post-treatment furcation involvement	Improved furcation involvement post-treatment	F1–F3	23	10/23	43.5%
			F1–F2	14	9/14	64.3%
			F3	9	1/9	11.1%
2. Subjective success	Subjective evaluation of pre- and post-treatment radiographs	Clinically improved	F1–F3	19	8/19	42.1%
3. Combined success	Comparison between 1 and 2	Objective and subjective success	F1–F3	9	2/9	22.2%

### Objective success (*n =* 23)

Post-treatment dental charts were available for 23 cases. There was objective furcation improvement in 43.5% (10/23) of the furcation defects treated with GTR (Table 3). This included 13.1% (3/23) of cases that were resolved and 30.4% (7/23) of cases that improved. In 21.7% (5/23) of cases, furcation involvement was static, and in 34.8% (8/23), there was no improvement in F3. This suggested that the furcation involvement did not worsen in any instance.

### Furcation involvement of 1 and 2 alone (*n =* 14)

When evaluating only those 14 teeth with either F1 or F2, there was objective furcation improvement in 64.3% (9/14) of the furcation defects treated with GTR. This included 21.4% (2/9) of cases that were resolved. Again, there were no instances of furcation involvement that worsened, suggesting that the 35.7% (5/14) of cases that were considered to have failed had static furcation involvement post-treatment.

### Furcation involvement of 3 alone (*n =* 9)

When evaluating the 9 teeth with F3, there was objective furcation improvement in only 11.1% (1/9) of cases. In that single case, F3 improved to F2. The remaining 88.9% (8/9) of cases showed no improvement and thus were considered to have failed.

An initial furcation involvement of F2 was significantly (*p* = 0.011) more likely to be successful (69.2%, 9/13) compared to an initial furcation involvement of F3 (11.1%, 1/9). No other factors were significantly associated with this outcome (Figure 10).

### Subjective success (*n =* 19)

Post-treatment dental radiographs were available for 19 cases. There was subjective furcation improvement in 42.1% (8/19) of the furcation defects treated with GTR (Table 3). No factors were significantly associated with this outcome (Figure 10).

## Discussion

This study found that in this canine cohort, GTR was clinically successful, defined as improvement in all evaluated parameters, in 90.3% (28/31) of vertical bone defects, and 22.2% (2/9) of furcation defects. This outcome was chosen to be the primary dictator of clinical success as it was the most clinically comprehensive and, accordingly, the least at risk of bias. However, to determine the true outcome of GTR, histology would be required.

Periodontal healing follows the general pathway of hemostasis, inflammation, proliferation, maturation, and remodeling. The ideal result is regeneration, with the new tissue structurally and functionally the same as the original tissue. For the periodontium, this means new cementum and alveolar bone with a periodontal ligament of functionally oriented collagen fibers (15). However, healing can also result in repair, with the new tissue structurally or functionally inferior to the original tissue (15, 16). For the periodontium, this can occur if there is (1) epithelialization of the internal face of the mucogingival flap so epithelial cells contact the root surface and form a long junctional epithelium; (2) connective tissue attachment that progresses to root resorption; or (3) bone growth with ankylosis and progression to root resorption (15, 16). There is likely a combination of regeneration and repair for any given site, with some areas of the site experiencing regeneration and others repair. The goal of GTR is to modulate healing in a way that promotes periodontal regeneration rather than repair. The aim of this study was to show the clinical success of GTR in maintaining teeth within the oral cavity in a large cohort of patients.

As there is no clear definition of “clinical success,” we elected to evaluate objective success based on change in CAL and VBDR, as well as subjective success based on the evaluation of radiographs by experts in the field. An agreement across these categories increased the confidence in the overall success and created a framework for the evaluation of GTR in future studies. However, it is prudent to recognize potential sources of unaccounted variation. Regarding attachment loss appreciated during the anesthetized oral exam, the penetration depth of a periodontal probe can vary between users, as well as with the presence and severity of periodontitis (17). Additionally, if the GTR site was remarked as normal, or there were no remarks regarding probing depth or gingival recession on anesthetized follow-up examination, it was assumed to be a normal gingival sulcus of maximal depth of 3 mm, though it could have been less. Regarding the change in bone height measured radiographically, all bone loss was accounted for as vertical bone loss by measuring the distance from the cementoenamel junction to the most apical aspect of bone loss at the GTR site and standardizing this measurement as a ratio to total root length, though there might have been a component of horizontal bone loss, which would not have been expected to improve with GTR treatment. All these potential sources of unaccounted variation would lead to under measuring the success of GTR.

Further, although teeth treated with GTR should always be evaluated with pre- and post-treatment periodontal probing and dental radiographs (18), there are cases in which there is post-treatment improvement in CAL but no post-treatment improvement in VBDR or vice versa. This is exemplified by the formation of a long junctional epithelium with improvement of CAL but no improvement of VBDR. This can still be a clinically important improvement resulting in stabilized periodontal disease and the tooth maintained within the oral cavity in a clinical patient. However, this is not considered a successful GTR since the periodontium was not regenerated. Therefore, we reported not only the combined clinical success rate of GTR but also described other objective and subjective success rates to help guide clinical decision-making.

We demonstrated a combined clinical success rate of 90.3% (28/31) for vertical bone defects treated with GTR. The median post-treatment improvement in CAL on oral examination was 1 mm. Although small, 1 mm represents enough attachment to allow the treated tooth to be maintained and therefore achieve a clinically relevant purpose. Additionally, there was a post-treatment improvement in CAL of 3 mm or greater in 32.1% (26/81) of cases, with a maximum improvement of 6 mm. The magnitude of post-treatment improvement in CAL was positively associated with the magnitude of initial CAL; thus, GTR may still be an appropriate treatment in cases with very deep periodontal pockets. There was also a 40% radiographic improvement in vertical bone pocket depth, which represents an improvement in the periodontal disease stage, as defined by the American Veterinary Dental College (14). It is interesting to note that the pre-treatment CAL was significantly greater for vertical bone defects at the veterinary center at the University of Minnesota than at the University of Wisconsin. Since this is a retrospective study, it is impossible to know the clinical decision-making in these cases and how this may have affected outcomes. There was no significant difference between the pre-treatment VBDRs at these veterinary centers.

The barrier membrane material was significantly associated with the outcome, with the bone membrane performing superiorly, especially with deeper bone defects. There was no identifiable difference between the initial lesions treated with different barrier membrane materials. However, it is possible that the outcome was impacted by different handling characteristics, which could have affected membrane placement quality by the surgeon and maintenance thereafter. The polymer membrane specifically begins as a liquid before undergoing a phase change to a solid. While the polymer membrane is indicated for the treatment and control of periodontal disease in dogs, it is not marketed as a barrier for guided tissue or/bone regeneration. However, GTR with the polymer membrane has been demonstrated clinically successful in dogs (9, 11). While the barrier membrane selection is often based on user preference, the polymer membrane is also a popular option in veterinary dentistry due to its economic cost. Other criteria for a barrier membrane include biocompatibility, barrier function with cell exclusion, tissue integration, handleability for clinical application, and stability during storage (11, 19). There are numerous barrier membrane options used in human dentistry that fall into the broader categories of non-resorbable, resorbable collagen-based, and resorbable synthetic (19). While non-resorbable membranes can provide significant mechanical support for space maintenance within the treated area, a second procedure is necessary for removal, making their use rare in veterinary dentistry (19). A resorbable collagen-based membrane is an alternative to the polymer and bone membranes in this study. While resorbable collagen-based membranes are biocompatible and have excellent cell affinity, these membranes are marketed for human use and, therefore, may incur a greater cost. Further, they are sourced from human, porcine, or bovine tissues, unlike the bone membrane, which is sourced from canine tissue (19). The polymer and bone membranes in this study are veterinary products with documented clinical success when used for GTR to treat naturally occurring periodontal disease in dogs (9–12).

Bone graft material was also significantly associated with the outcome, with alloplasts performing superiorly. While the barrier membrane is the material that excludes gingival epithelial and connective tissue downward migration into the site essential to GTR, different bone graft materials variably display the different properties of osteogenesis with the presence of osteoprogenitor cells, osteoinduction largely due to bone morphogenic protein content, and osteoconduction as a scaffold (20, 21). While autografts are osteogenic, osteoinductive, and osteoconductive, allografts are osteoinductive, and osteoconductive, and alloplasts are only osteoconductive (20, 21). Therefore, one may expect a more robust regenerative response with the use of autografts and allografts, which should cause enhanced tissue ingrowth compared with alloplasts. Surprisingly, alloplasts performed superiorly. This was the case even though alloplasts were used in only 6 of the 53 cases evaluated, autografts in 9 cases, and allografts in 38 cases. This may stem from the fact that the presence of a scaffold to maintain the position of the membrane is the most important for GTR success.

GTR was used to successfully treat vertical bone defects of many teeth and in many locations around the treated teeth, supporting that any tooth with a deep infrabony pocket is a candidate for GTR. However, with fewer walls, there is likely decreased stability of the bone graft to maintain space for clot stabilization and support the overlying barrier membrane without displacement, though this was not evaluated in this study. There was also no significant association of outcome with the extraction of a tooth adjacent to the GTR site. However, an adjacent tooth was extracted for 83.3% (20/24) of mandibular first molars but only for 30.8% (4/13) of maxillary canines. Crowding may occur more frequently for the mandibular first molar with the mandibular fourth premolar and mandibular second molar, compared with the maxillary canine and its surrounding teeth. Such crowding may not only predispose to rapid progression or severe manifestation of periodontal disease but also physically interfere with GTR execution, resulting in adjacent tooth extraction. Clinical judgment is needed to determine when adjacent tooth extraction is necessary or otherwise beneficial. Additionally, it is important to consider the pulp status when recommending GTR. Severe periodontal disease can result in secondary pulpitis. In fact, a study that focused on histological evaluation of teeth with moderate to advanced periodontal disease (majority periodontal disease stage 4) found pulp necrosis in 9/22 (40.9%) of the teeth, and acute or chronic pulpitis in 6/22 (27.3%) of the teeth (22). Pulp necrosis was thought to be due to the high incidence of tooth mobility compromising the blood supply, but advanced periodontal disease did seem to also influence the pulp (22). While histology was not evaluated in the current study, there was no evidence of tooth non-vitality, or lesions of endodontic origin noted radiographically for any tooth treated with GTR in the current study.

Aside from regeneration, GTR can result in periodontal repair with stabilized periodontal disease. While this was not considered a combined success, this can be a clinically important improvement for the patient and therefore was described in this study as other objective and subjective success rates. There was objective success in 88.6% (31/35) of vertical bone defect cases. However, there were an additional 14 cases for which there was no consensus for change in CAL and VBDR, indicating no objective consensus, be that success or failure. Of those without an objective consensus, 64.3% (9/14) had improvement in VBDR but a change in CAL equal to zero. In this scenario, a change in CAL equal to zero could have been seen with static gingival recession with no change in the sulcus or pocket depth or with persistence or recurrence of a periodontal pocket despite a gain in vertical bone height. Additionally, of those without an objective consensus, 14.3% (2/14) had improvement in CAL but a change in VBDR equal to zero. In this scenario, a change in VBDR equal to zero could be due to periodontal repair with a long junctional epithelium rather than regeneration. While we categorized these 11 cases as failed for the purposes of this study, since they did not meet the stringent consensus criteria for objective success, the result of those 11 cases is expected to have been clinically acceptable for maintaining the treated tooth in the oral cavity. While successful GTR results in periodontal attachment gains, failed GTR can still result in the stagnation of periodontal disease and does not force exodontia in all instances.

There was subjective radiographic improvement in 90.7% (49/54) of vertical bone defect cases, confirming that in many cases, despite stagnant periodontal probing depths, clinicians would term the GTR of many teeth as successful and maintain the teeth within the oral cavity.

Unlike GTR for vertical defects, GTR had poor success when used in furcation defects, with a combined clinical success rate of approximately 22.2% (2/9). However, lesion-specific success rates were 64.3% (9/14) for F1 and F2 lesions combined and 11.1% (1/9) for F3 lesions. This is not surprising, as again, with fewer walls, there is decreased stability of the bone graft to maintain space for clot stabilization and support the overlying barrier membrane without displacement. Zacher and Marretta (23) recently provided a comprehensive review of furcation lesions, including emphasizing characteristics of teeth with furcation lesions that are favorable for maintaining those teeth in the oral cavity. In the context of GTR, a wide furcation entrance area with wide root divergence may be favorable because these characteristics facilitate detection and access for treatment of furcation defects (23). The current study confirmed that GTR in F3 lesions is highly likely to fail. Therefore, we advocate for the judicious use of GTR to treat F1 and F2 lesions with proper client counseling.

Of note, 57.1% (80/140) of patients that had GTR performed were lost to follow-up. For the mandibular first molar and maxillary canine, which were the most common teeth treated with GTR for vertical bone defects, severe complications of unsuccessful GTR treatment include pathologic mandibular fracture and development of oronasal fistula, respectively. Since GTR was not demonstrated to be 100% clinically successful, we generally advocate for stringent patient and client selection when offering GTR. Considerations include current daily at-home dental care, history of routine annual professional dental cleanings, and progressive comorbidities that might affect future anesthetic events.

The findings from this study should be evaluated in the context of its limitations, notably the retrospective nature of this study and lack of standardized care amongst all cases. Not only does this study bridge two institutions, but also multiple residents and board-certified specialists. There were no standardized materials or protocols, and surgical experience likely influenced outcomes. Second, not all information was present in the available medical records. Particularly, a complete set of anesthetized oral examination charts and diagnostic radiographs were not available for all patients. Additionally, no specific complications were noted, such as dehiscence and membrane exposure. Finally, we reiterate that we evaluated only the clinical success of GTR without histology to investigate true tissue regeneration.

Furthermore, the clinical success of GTR was evaluated without comparison to root planing alone. It is standard to perform RP/O at the time of GTR. However, our data was not directly compared to teeth that received RP/O or closed root planing (RP/C) alone. Based on human standards, RP/C is recommended for pockets up to 5 mm, and a flap is recommended for deeper pockets (24).

When considering the 81 cases included for objective attachment gain analysis in this study, approximately half (43/81) had a pre-treatment CAL ≤ 5 mm. Due to the retrospective nature of the study, clinical decision-making to perform GTR vs. RP/C is unknown. Furthermore, the difference in clinical improvement that may have been gained between GTR and RP/C is unknown.

Based on our data set, we were able to estimate improvements for a pre-treatment CAL of 4 mm and 8 mm. Since there was a significant difference in improvement between the polymer and bone barrier membranes, the model also accounted for this factor. For animals with a pre-treatment CAL of 4 mm, we estimate an improvement of 0.04 ± 2.83 mm with a polymer membrane and 1.11 ± 2.85 mm with a bone membrane, while for animals with a pre-treatment CAL of 8 mm, we estimate an improvement of 3.24 ± 2.87 mm with a polymer membrane, and 4.3 ± 2.86 mm with a bone membrane (Table 4).

**Table 4:** Comparison of GTR treatment results for vertical bone defects from the current study with RP/C, RP/O, and RP/O with implant placement results from two other veterinary studies (25, 26).

**Treatment**	**Initial probing depth (mm)**	**Improvement (mm)**	**Study**
RP/C	3.5	0.8 ± 0.6	25
	4.0–4.5	1.4 ± 0.8	
	5.0–5.5	2.1 ± 0.5	
RP/O	3–8	1.0 (0–4)	26
RP/O with implant placement	4–8	2.0 (0–4)	26
GTR with polymer membrane	4	0.04 ± 2.83	Current study
	8	3.24 ± 2.87	
GTR with bone membrane	4	1.11 ± 2.85	Current study
	8	4.3 ± 2.86	

Comparatively, Martel et al. (25) evaluated RP/C to treat 21 teeth in 10 dogs with initial periodontal pocket depths of 3.5–5.5 mm. Twelve weeks following treatment, there was an average improvement of 1.5 ± 0.8 mm (Table 4). While this does not provide a direct comparison, especially as follow-up time differed, these results are similar to our estimated improvement of a pre-treatment CAL of 4 mm treated with GTR with a bone membrane and greater than our estimated improvement with a polymer membrane. Therefore, RP/C, which is the more conservative option, maybe a superior option for minimal vertical defects.

In another prospective study utilizing a split-mouth model, nine dogs received RP/O alone and RP/O with placement of an implant of medical grade porcine gelatin cross-linked by transglutaminase into a porous scaffold to treat 22 teeth with periodontal disease stage 2 to early stage 4 with initial probing depths 3–8 mm (26). Three months following treatment, teeth treated with RP/O alone had an average (range) improvement in probing depth of 1.0 mm (0–4 mm) (Table 4). Teeth treated with RP/O with implant placement had an average (range) improvement in probing depth of 2.0 mm (0–4 mm), which was a significant improvement compared with RP/O alone (Table 4).

Again, while direct comparisons are limited due to pointed differences in study design, these results are less than our estimated improvement of a pre-treatment CAL of 8 mm treated with GTR with both bone and polymer membranes. Additionally, furcation involvement remained largely static in all cases for both groups in the Gawor et al. (26) study, which included F1, F2, and F3 lesions. These results are less than our findings of 64.3% improvement or resolution for F1 and F2 lesions and similar to our findings of 11.1% improvement for F3 lesions treated with GTR. Controlled prospective studies are indicated to directly compare RP/C, RP/O, and GTR treatments to counsel clinical decision-making. Other areas of improvement for future studies include utilizing cone beam computed tomography rather than dental radiography for more precise quantitative measurements of vertical bone defect depth, total vertical bone defect volume, and furcation involvement (27).

Despite its limitations, this is the largest study to date to report on the clinical success of GTR in dogs. It was found that 90.3% (28/31) of vertical bone defects improved, and 64.3% (9/14) of F1 and F2 furcation defects improved. F3 lesions treated with GTR had a high failure rate of 88.9% (8/9). The bone membrane appears superior to the polymer membrane, and alloplasts appear superior to allografts or autografts when a bone graft material is used. GTR is associated with a high level of clinical success and is an appropriate treatment to maintain teeth in the oral cavity of dogs, given appropriate client and patient selection.

## Data availability statement

The raw data supporting the conclusions of this article will be made available by the authors, without undue reservation.

## Ethics statement

Ethical review and approval was not required for the animal study because the study is retrospective in nature and included clinical cases; hence, it is exempt from IACUC requirements. Written informed consent for participation was not obtained from the owners because the study is retrospective in nature; hence, it is exempt from requiring written informed consent.

## Author contributions

BL, JS, and SG: project conception and design and radiograph analysis. BL: data acquisition. AR: statistical analysis. BL and SG: initial manuscript draft. All authors contributed to the manuscript revision, read, and approved the submitted version.
