# Supplementary material for: Clinical success of guided tissue regeneration for treating vertical bone and furcation defects in dogs
Source: Front Vet Sci. 2023 Aug 30;10:1247347. doi: 10.3389/fvets.2023.1247347 (PMC10498771; doi:10.3389/fvets.2023.1247347)
Supplement: Supplementary file 1 [file Table_1.pdf]

Supplemental Table 1: Publications of GTR in dogs, including case reports, case series, and a prospective study. Clinical success was documented in all instances, providing proof-of-concept.

Publication	Tooth/Teeth	Pre-operative degree of periodontal disease	Bone graft material	Barrier membrane	Post-operative awake recheck at 10-14 days	Post-operative anesthetized recheck at 6-8 months
Smith, 1995<br><br>Case report (10 year-old German Shepherd dog)	-Chronic periodontal defect secondary to a string foreign body in the interproximal space of left mandibular fourth premolar and first molar involving soft tissue and bone	-4mm gingival recession with a 6mm periodontal pocket for 10mm of total attachment loss<br>-Radiographs revealed periodontal bone loss	Autogenous corticocancellous bone harvested from the left ventral mandible	None	-No post-operative complications reported by the veterinarian owner, but not rechecked by the clinician	-4mm gingival pocket<br>-Partial restoration of periodontal bone with a persistent 4-5mm bone defect resulting in approximately 60% diminution in the size of the original boney defect
Beckman, 2004<br><br>Case report (7 year-old female spayed American Eskimo dog)	-Right mandibular first molar	-6mm periodontal pocket on the distal aspect<br>-Vertical 2-walled osseous defect on the distal aspect of the distal root with a 2-mm decrease in alveolar crestal bone height	Consil	None	-Complete healing of the surgical site	-3mm periodontal pocket on the distal aspect<br>-Bone deposition on the distal aspect with a 1mm infrabony defect measured radiographically<br>-Restoration of the periodontal space
Rice, Snyder, and Soukup, 2012<br><br>Case report (11 year-old male castrated Cairn Terrier)	-Left mandibular first molar (mesial aspect of the mesial root)	-12mm periodontal pocket along mesial root<br>-Approximately 50% vertical bone loss associated with only the mesial root<br>-2-walled infrabony defect of the mesial root	Autogenous cortical bone harvested from the vestibular cortical plate ventral to the alveolar bone of right mandibular first molar	Doxirobe	-Recommend to be performed by referring veterinarian due to travel distance - no further remarks	-2mm gingival recession with 2mm attached gingiva, for 2mm total attachment loss<br>-Radiographically appropriate alveolar crestal bone height with osteointegration of graft<br>-Radiographically apparent PDL space traceable along the entire length of the mesial root surface
Stepanuik & Gingerich, 2015	-Right maxillary fourth premolar (x1)	-The mean periodontal probing depth was 7.2- mm (range 5 to 12-	Periomix	Ossiflex	-No membrane exposure, evidence of surgical site infection,	-All periodontal probing depths decreased to < 3-mm, with a mean

Prospective study (11 teeth in 8 client owned dogs)	-Left maxillary fourth premolar (x2)<br>-Right maxillary canine (x5)<br>-Left maxillary canine (x3)	mm, median 6-mm)<br>-Intrabony pockets present in all cases			hemorrhage, or swelling, and the periodontal flaps were healed	depth gain of 5.4mm<br>-Intrabony bony pocket depth decreased in all cases<br>-Intraoral radiographic improvement of alveolar bone height was subjectively noted in all cases
Alterman & Huff III, 2016<br><br>Case series (4 teeth in 3 client owned dogs)	-Right maxillary canine (x1)<br>-Left maxillary canine (x2)<br>-Left mandibular first molar (x1)	-Stage 2-4 periodontal disease	Periomix	Doxirobe	-Complete healing of all GTR sites	-Attachment gain and new bone growth of all GTR sites
Bebel & Rochette, 2019<br><br>Case report (10 year-old female spayed miniature dachshund)	-Left mandibular canine (lingual aspect)	-12mm lingual periodontal pocket<br>-3-walled bony pocket on the lingual aspect<br>-Stage 3 periodontal disease	Periomix	Ossiflex	-Surgical site healing	-Periodontal probe measurement 3mm lingually<br>-Radiographic resolution of the infrabony pocket
